# Icariside II Exerts Anti-Type 2 Diabetic Effect by Targeting PPARα/γ: Involvement of ROS/NF-κB/IRS1 Signaling Pathway

**DOI:** 10.3390/antiox11091705

**Published:** 2022-08-30

**Authors:** Yiqi Li, Yeli Li, Nana Chen, Linying Feng, Jianmei Gao, Nan Zeng, Zhixu He, Qihai Gong

**Affiliations:** 1 School of Pharmacy, Chengdu University of Traditional Chinese Medicine, Chengdu 611137, China; 2 Key Laboratory of Basic Pharmacology of Ministry of Education and Joint International Research Laboratory of Ethnomedicine of Ministry of Education, School of Pharmacy, Zunyi Medical University, Zunyi 563000, China; 3 The Collaborative Innovation Center of Tissue Damage Repair and Regeneration Medicine, Zunyi Medical University, Zunyi 563000, China

**Keywords:** Icariside II, Type 2 diabetes mellitus, peroxisome proliferator-activated receptors α and γ, oxidative stress, inflammation

## Abstract

Type 2 diabetes mellitus (T2DM) is a multisystem and complex metabolic disorder which is associated with insulin resistance and impairments of pancreatic β-cells. Previous studies have shown that icariside II (ICS II), one of the main active ingredients of *Herba Epimedii*, exerts potent anti-inflammatory and anti-oxidative properties. In this study, we investigated whether ICS II exerted anti-T2DM profile and further explored its possible underlying mechanism both in vivo and in vitro. db/db mice were administered ICS II (10, 20, 40 mg·kg^−1^) for 7 weeks. We found that ICS II dose-dependently attenuated hyperglycemia and dyslipidemia, as well as inhibited hepatic steatosis and islet architecture damage in db/db mice. Moreover, ICS II not only dramatically reduced inflammatory cytokines and oxidative stress, but also up-regulated PPARα/γ protein expressions, phosphorylation of Akt, GSK3β and IR, meanwhile, down-regulated phosphorylation of NF-κB(p65) and IRS1 in db/db mice. In palmitic acid (PA)-treated HepG2 or MIN6 cells, ICS II (5−20 μM) concentration-dependently promoted the cell viability via mediating PPARα/γ/NF-κB signaling pathway. PPARα/γ knockout by CRISPR-Cas9 system partly abolished the protective effects of ICS II on HepG2 or MIN6 cells following PA insults. These findings reveal that ICS II effectively confer anti-T2DM property by targeting PPARα/γ through mediation of ROS/NF-κB/IRS1 signaling pathway.

## 1. Introduction

Type 2 diabetes mellitus (T2DM) is one of the most grievous chronic diseases and is an accumulatively significant public health problem and challenge worldwide, despite the availability of multiple treatments [1,2]. Currently, therapies for T2DM are limited in clinics due to adverse drug effects (especially weight gain and hypoglycemia) or contraindications [3,4]. Thus, more safe and effective drugs to treat T2DM are still in dire need.

The development of T2DM is characterized by a decrease in insulin sensitivity, also called insulin resistance, resulting in hyperglycemia [5]. Pathogenesis of T2DM and development of insulin resistance are evoked by multiple stimuli factors, notably the occurrence of oxidative stress and inflammation [6,7]. Oxidative stress occurs in T2DM as a result of excessive generation of reactive oxygen species (ROS), which can elicit insulin resistance and signaling pathway in insulin-responsive tissues, as well as affect the development of various diabetic complications [8]. Besides, excessive ROS can also activate nuclear factor-kappaB (NF-κB) and then promote release of pro-inflammatory cytokines such as interleukin (IL)-1β, IL-6, tumor necrosis factor (TNF)-α [9]. These pro-inflammatory cytokines injure the insulin signaling and trigger insulin resistance, even aggravating ROS generation [10,11]. Thus, attenuation oxidative stress and inflammation is a promising strategy to conquer T2DM. Emerging evidence suggests that peroxisome proliferator-activated receptors (PPARs), ligand-inducible transcription factors, which mediate multiple genes expression, are involved in adipogenesis, oxidative stress, and inflammation [12,13]. A great deal of metabolic pathways obsessed in T2DM are regulated by PPARs [14,15]. PPARs belong to the nuclear receptor superfamily and have been classified into three isoforms, including PPARα, PPARβ, and PPARγ. Among them, PPARα is mainly located in the liver, where it mediates the adaptive response to fasting through controlling fatty acid transport, β-oxidation, and ketogenesis [16,17]. PPARγ is mainly enriched in adipose tissues, where it mediates the mature fat storing and enhances insulin sensitivity and lipogenesis [18]. Therefore, PPARα/γ are potential therapeutic targets for treatment of T2DM, and dual PPARα/γ agonist is a potent agent to overcome T2DM. However, it is regrettable that existing PPARα/γ agonists have not been used in clinics due to their severe adverse drug effects, such as inducing cancer [19,20]. Thus, developing new PPARα/γ agonists with adequate anti-diabetic efficacy and a safe profile is of great significance to T2DM treatment.

Fortunately, lead compounds and new drugs deriving from natural products exhibit the merits of high efficiency and good security. Icariside II (ICS II) is derived from *Herba Epimedii* which possesses multiple pharmacological effects, including anti-cancer [21], anti-inflammatory [22,23], and antioxidative activities [24,25]. Of note, our previous studies found that ICS II effectively alleviated cognitive impairments induced by chronic cerebral hypoperfusion and protected against cerebral ischemia-reperfusion injury via up-regulating the protein expressions of PPARα and PPARγ [26,27]. However, it remains unknown whether ICS II can exert beneficial on T2DM via activating PPARα/γ. Therefore, this study was designed to investigate the effect and detailed mechanism of ICS II against T2DM in db/db mice in vivo and palmtic acid (PA)-induced injury in HepG2 cells and MIN6 cells in vitro.

## 2. Materials and Methods

### 2.1. Experimental Design

All animal care and experiment protocols were performed in compliance with international guidelines for care and use of laboratory animals published by the US National Institutes of Health (National Institutes of Health Publication 85-23, revised 1996), and were approved by the experimental animal ethics committee of the Zunyi Medical University (Guizhou, China). 6-week-old Male C57BLKS/Leprdb (db/db) mice and littermate C57BLKS wild-type (WT) mice were purchased from the model animal research centre of Nanjing University. Icariside II (purity ≥ 98%) was purchased from Nanjing Zelang Medical Technology Corporation Ltd. (Nanjing, China). Metformin (Met) was purchased from Sino-American Shanghai Squibb Pharmaceuticals Ltd. (Shanghai, China). The animals were housed at 23 ± 1 °C, 55 ± 5% relative humidity and subjected to a regular 12 h/12 h light/dark cycle throughout the experimental period, allowing free access to a normal chow diet and water. After 1 week of acclimatization, plasma glucose levels of all mice were assessed to verify the diabetic status, and a total of 40 db/db mice were randomly divided into five groups (*n* = 8/group): diabetic model group (db/db, treated with normal saline), low-dose ICS II-treatment group (ICS II, 10 mg·kg^−1^), medium-dose ICS II-treatment group (ICS II, 20 mg·kg^−1^) and high-dose ICS II-treatment group (ICS II, 40 mg·kg^−1^), Met -treatment group (Met, 100 mg·kg^−1^), age-matched male WT mice (treated with normal saline) were used as the normal control (*n* = 8/group), the other WT mice were treated with ICS II (40 mg·kg^−1^) alone (*n* = 8/group), and ICS II or vehicle was administered to mice via oral gavage twice a day. Fasting blood glucose levels and body weight were monitored weekly. At the end of treatment, mice were fasted overnight and deeply anaesthetized with an overdose of isoflurane. Blood samples were collected through the venous plexus behind the eyeball, and tissues were immediately collected until further analysis.

### 2.2. Oral Glucose Tolerance Test and Insulin Tolerance Test

For the oral glucose tolerance test (OGTT), mice were performed by glucose gavage (2 g·kg^−1^) after starvation for 12 h, and blood glucose levels were collected by pricking the tail vein at 0, 15, 30, 45, 60, 90, and 120 min after administration [28]. For intraperitoneal insulin tolerance test (IPITT), six-hour fasted mice were injected intraperitoneally with insulin (0.75 U/kg), blood glucose levels were measured sequentially from the tail vein at 0, 15, 30, 45, 60, 90, and 120 min after injection, and the area under curve (AUC) was calculated from the data collected during the OGTT and IPITT experiments. 

### 2.3. Microarray Processing and Data Analysis

Total RNA was extracted from the liver tissues of mice in WT, db/db, and db/db + ICS II (40 mg·kg^−1^) groups using TRIzol buffer on the basis of experimental protocol. RNA samples were detected based on the A260/A280 absorbance ratio with a Nanodrop ND-2000 system (Thermo Scientific, Waltham, MA, USA), and the RIN of RNA was determined by an Agilent Bioanalyzer 4150 system (Agilent Technologies, Santa Clara, CA, USA). Only qualified samples will be used for library construction. Sequencing was performed with an Illumina Novaseq 6000 /MGISEQ-T7 instrument. The data generated from Illumina/BGI platform were used for bioinformatics analysis. FeatureCounts (http://subread.sourceforge.net/, accessed on 7 May 2021) was used to count the reads numbers mapped to each gene. Then, the FPKM of each gene was calculated based on the length of the gene and reads count mapped to this gene. Gene expression with a fold change (FC) greater than 1.5 and *P*-value less than 0.05 were identified as differentially expressed genes (DEGs) in this study. The enrichment of pathways and functional processing were formed based on Kyoto Encyclopedia of Genes and Genomes (KEGG) and annotations of Gene Ontology (GO) (http://www.genome.jp/kegg/pathway.html, accessed on 7 May 2021).

### 2.4. Immunohistochemistry

Pancreas from all mice were collected and fixed in 4% formaldehyde solution overnight. Subsequently, paraffin-embedded pancreas sections were rehydrated and heated with sodium citrate buffer for antigen retrieval and then blocked with 10% goat serum to block endogenous peroxidases. Next, the sections were incubated with anti-insulin antibody (1:1000; Abcam, Cat# 181547) overnight at 4 °C. For color development, we used the HRP-DAB system, and then captured them as digital images.

### 2.5. Cell Culture and Drug Treatment

HepG2 cells and MIN6 cells (secondary generation cells, ATCC, Manassas, VA, USA) were respectively cultured in Dulbecco’s Modified Eagle’s Medium (DMEM) and Roswell Park Memorial Institute (RPMI)-1640 containing 10% fetal bovine serum and 1% penicillin/streptomycin at 37 °C in a humidified atmosphere containing 5% CO_2_. The cells were generally seeded into six-well plates at 1 × 10^5^ cells per well, and at 70% confluence, the growth medium was changed to DMEM or RPMI-1640 containing 300 μM PA. Next, cells were treated with various concentrations of ICS II (5, 10, 20 μM) or saroglitazar (Saro, a postive control drug, 40 μM) for 24 h.

### 2.6. Determination of Cell Viability

Cells were treated as described above, after 24 h treatment, 3-4, 5-dimethylthiazol -2, 5-diphenyltetrazolium bromide (MTT) was added to each well at an ultimate concentration of 5 mg·mL^−1^ for another 4 h. Thereafter, the medium was then removed and 150 μL DMSO was added to dissolve the formazan. Next, the absorbance of formazan was detected at 490 nm using a microplate reader. Cell viability was expressed in percentage of the value of the control group.

### 2.7. ELISA Assay 

The tissues and cells were collected and homogenized using 0.1 M PBS (pH 7.4). The resulting suspension was subjected to freeze–thaw cycles to further break the cell membranes. Blood samples were allowed to clot for 2 h at room temperature. Thereafter, the homogenates were centrifugated for 20 min at 1000× *g*. Then, the supernatant was removed and determined using related ELISA kits according to the manufacturer’s protocol.

### 2.8. DHE Staining and Mito-Sox Staining

Flash-frozen livers and pancreas were sectioned at 7 μm and incubated with 10 μM DHE for 30 min. HepG2 cells were seeded in six-well plates and treated as previously. After being rinsed thrice with PBS, cells were incubated with 5 µM Mito-Sox for 10 min. Fluorescence emission was detected using fluorescence microscopy. 

### 2.9. Oil Red O Staining

The liver cryosections or HepG2 cells were rinsed three times in PBS and fixed in 4% paraformaldehyde for 10 min and then stained with Oil Red O solution for 10 min at room temperature. After this, sections were washed with PBS to remove unbound dye and nuclei were counter-stained with hematoxylin for 1 min. Representative images were photographed with an Olympus light microscope fitted with a digital camera. ImageJ software was used to evaluate the degree of steatosis.

### 2.10. RNA Interference 

For RNA interference experiments, HepG2 cells and MIN6 cells were randomly plated onto 6-well plates at 1 × 10^5^ cells per well in DMEM or 1640. Lentiviral vector and virus packaging have been completed by Hanheng Biotechnology (Shanghai, China) Co., Ltd. The general procedures are plasmid construction, transfection of 293T cells, output, collection and purification, and detection titer. The PPARα-targeted lentivirus and PPARγ-targeted lentivirus were diluted with Opti-MEM and balanced for 15 min at room temperature. Next, cells were transfected with PPARα and PPARγ lentivirus following the manufacturer’s protocol. The PPARα/γ gRNA sequences are as follows (Table 1).

### 2.11. Protein Quantification and Western Blot Analysis

For western blot analysis, tissues and cells were homogenized with fresh RIPA buffer added with phenylmethanesulfonyl fluoride (100:1) and phosphatase inhibitor (100:1). The total protein concentrations of the lysates were determined by using a BCA kit. Membrane proteins were obtained using a membrane protein extraction kit. The lysates were normalized to equal amounts of protein, and 30 micrograms protein from tissue lysates or 20 micrograms protein from cell lysates were subjected to sodium dodecyl sulfate polyacrylamide gel and transferred to an immune-blot PVDF membrane, which was blocked for 2 h in 5% skim milk and incubated with primary antibodies against overnight at 4 °C, GLUT4 (1:2,000; Abcam, Cat# 645), p-Akt (1:1,000; Abcam, Cat# 131443), Akt (1:1,0000; Abcam, Cat# 179463), p-GSK3β^Ser 9^ (1:1,000; Abcam, Cat# 131097), GSK3β (1:1,000; Abcam, Cat# 93926), p-IRS1^Ser 307^ (1:1,000; Abcam, Cat# 5599), IRS1 (1:1,000; Abcam, Cat# 52167), p-IR^Tyr 1185^ (1:1,000; Abcam, Cat# 203278), IR (1:500; Abcam, Cat# 203746), p-IKKβ (1:1,000; Abcam, Cat# 194519), IKKβ (1:1,000; Abcam, Cat# 124975), p-NF-κB(p65) (1:1,000; Abcam, Cat# 76302), NF-κB(p65) (1:1,000; Abcam, Cat# 16503), IκB-α (1:1,000; Abcam, Cat# 32518), PPARα (1:1,000; Abcam, Cat# 215270), PPARβ (1:1,000; Abcam, Cat# 17866), PPARγ (1:1,000; Abcam, Cat# 227074), anti-β-tubulin (1:20,000; Proteintech, Cat# 66240-1-lg), and anti-β-actin (1:5000; Proteintech, Cat# 60008-1-Ig). Thereafter, membranes were incubated with secondary antibodies for 1 h at room temperature and were performed using the enhanced chemiluminescence system, and Image Lab software to quantify the band optical intensity. To control for unwanted sources of variation, the relative expression of target proteins in various groups were normalized to that evaluated for β-actin or β-tubulin and were expressed as the relative fold to those of the WT or control group.

### 2.12. Statistical Analysis

The data and statistical analysis comply with the recommendations on experimental design and analysis in pharmacology. All data were presented as mean ± SEM, and two or more groups were compared by Student’s t-test or one-way ANOVA by using SPSS 22.0. Graphs were drawn by GraphPad Prism 8.0. Values of *p* < 0.05 were considered to be a statistically significant difference.

## 3. Results

### 3.1. ICS II Prevented Hyperglycemia and Restored the Structural Integrity of the Pancreas in db/db Mice

**Figure 1:**
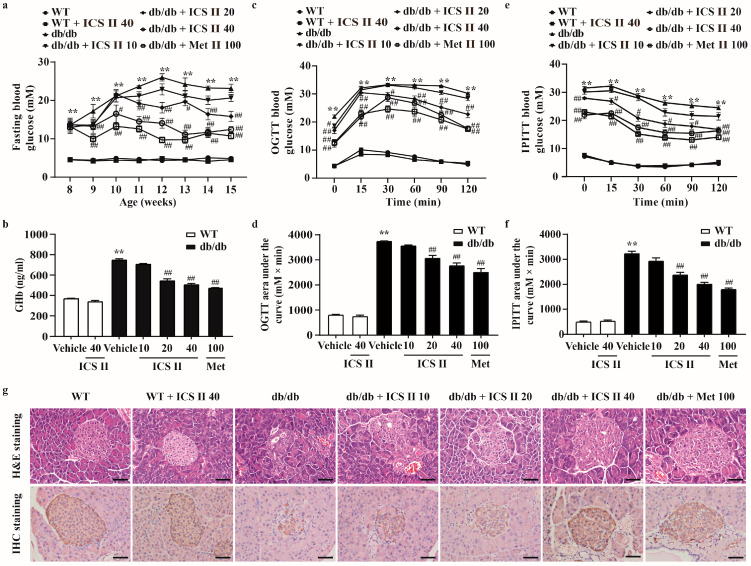
ICS II improved glucose metabolism, insulin sensitivity and pancreatic islet architecture in db/db mice. ICS II at doses of 10, 20, or 40 mg·kg^−1^ was administered to db/db mice or WT mice (starting from 8 weeks old) for 7 weeks. (**a**) 12 h fasting blood glucose of the mice (*n* = 8). (**b**) GHb level (*n* = 8). OGTT (**c**,**d**) and IPITT (**e**,**f**) were performed after 6 weeks of ICS II administration and the AUCs were calculated (*n* = 8). (**g**) H&E staining and insulin immunostaining (×400, scale bar = 50 μm). Data were expressed as mean ± SEM. ** *p* < 0.01 versus WT group; ^#^ *p* < 0.05 and ^##^ *p* < 0.01 versus db/db group.

To investigate whether ICS II could decrease the high blood glucose level of db/db mice, the fasting blood glucose was tested. The results showed that fasting blood glucose of db/db mice was dramatically increased, more than that of the WT mice. However, ICS II (20 and 40 mg·kg^−1^) and metformin (Met) effectively lowered fasting blood glucose levels. Of note, ICS II alone did not affect the fasting blood glucose of WT mice (Figure 1a). These findings suggested that ICS II significantly reduced the blood glucose level with safety profile. Glycosylated hemoglobin (GHb), which reflects long-term glycemic control, was also significantly decreased after ICS II treatment compared with the db/db group (Figure 1b). OGTT and IPITT assays were respectively used to evaluate the glucose tolerance and insulin sensitivity. The AUC of OGTT and IPITT was reduced in the ICS II (20 and 40 mg·kg^−1^) group compared with that in the db/db group, which showed that ICS II improved glucose clearance, and significantly decreased insulin resistance in db/db mice (Figure 1c–f). There were no significant changes in body weight among treatment groups and db/db group (Supplementary Figure S1a). Moreover, the pancreas was isolated from each experimental group and analyzed by H&E staining or immunostaining with anti-insulin antibody. The results showed that pancreatic islet architecture clearly differed between the WT and db/db groups. In WT mice, the islets harbored tight, insulin positive beta cells, whereas the islets in db/db mice were enlarged and disorganized with diffusion into the surrounding tissue. Notably, ICS II treatment significantly increased the number of β cells. More importantly, many insulin-positive cells displayed a relatively dense arrangement compared with those in the db/db mice (Figure 1g). These findings indicate that ICS II effectively rescues T2DM in db/db mice. 

### 3.2. ICS II Mitigated Hepatic Steatosis and Dyslipidemia in db/db Mice

**Figure 2:**
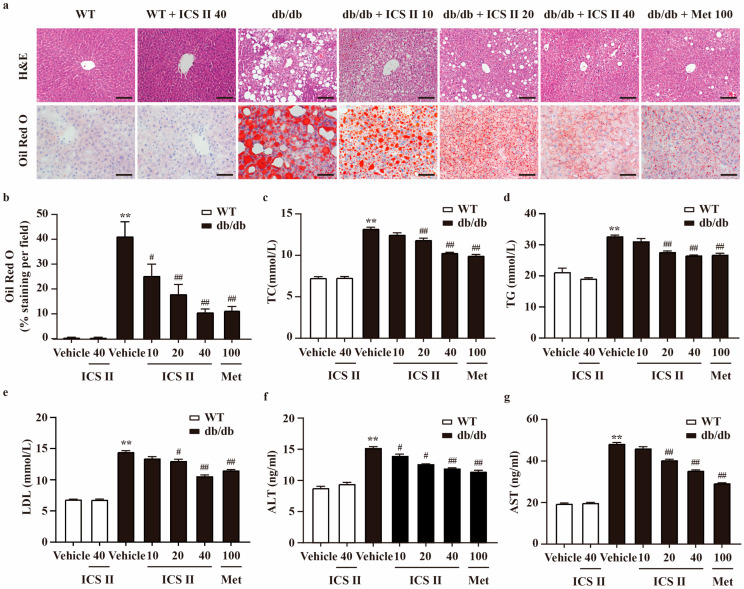
ICS II improved lipid profiles in the serum and hepatic steatosis of db/db mice. (**a**) H&E staining (×200, scale bar = 100 μm) and Oil red O staining (×400, scale bar = 50 μm). (**b**) Quantitation of Oil red O staining. The area of positive staining per field were calculated using ImageJ. (**c**) TC level (*n* = 8). (**d**) TG level (*n* = 8). (**e**) LDL level (*n* = 8). (**f**) ALT level (*n* = 8). (**g**) AST level (*n* = 8). Data were presented as mean ± SEM. ** *p* < 0.01 versus WT group; ^#^ *p* < 0.05, ^##^ *p* < 0.01 versus db/db group.

Liver HE staining illustrated severe fatty degeneration hepatocytes, and numerous vacuolated and swollen hepatocytes in db/db mice. However, ICS II significantly alleviated vesicular steatosis and ballooning in the liver of db/db mice. Moreover, Oil Red O staining of frozen liver sections further showed that hepatic lipid accumulation was decreased in ICS II treated db/db mice, manifested as relatively lighter red, compared with model control showing many lipid droplets in deep red (Figure 2a,b). In line with the hepatic lipid accumulation in the db/db model, liver weight of db/db mice was higher than that of WT mice, and ICS II significantly decreased the values of the index (Supplementary Figure S1b). To further explore the effect of ICS II on lipid metabolism, several blood parameters were analyzed. In db/db mice, serum total cholesterol (TC), triglycerides (TG) and LDL-cholesterol were obviously higher than that of WT mice. In ICS II-treated mice, these parameters were pronouncedly reduced (Figure 2c–e). Similarly, ICS II evidently alleviated high levels of plasma ALT and AST in db/db mice (Figure 2f,g). These results suggest that ICS II mitigates hepatic steatosis, hyperlipidemia, and liver damage. 

### 3.3. Microarray Data Analysis

**Figure 3:**
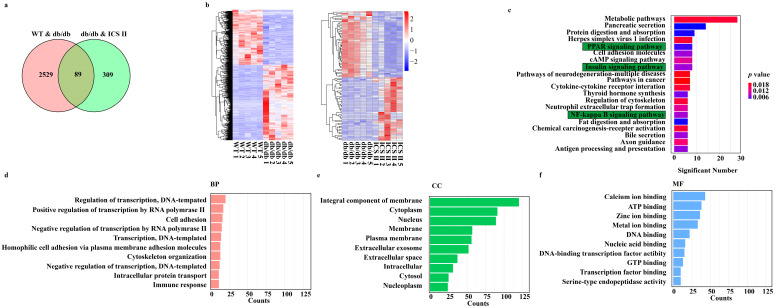
Analysis of DEGs profiling of the liver in mice. (**a**) The DEGs between WT versus db/db groups and ICS II versus db/db groups were summarized in a Venn diagram. (**b**) The correlation coefficients between altered gene expression profiles were analyzed using hierarchical clustering analysis. (**c**) Top KEGG pathways enriched with DEGs and their matching *P* values. (**d**–**f**) Bar graph shows the fold enrichment values of the top 10 most significantly enriched terms for the regulation of genes, analyzed by GO terms.

DEGs were determined in the condition of both *p* value < 0.05 and FC > 1.5. As shown in Figure 3a, there were 2618 overlapped DEGs between WT versus db/db, 398 overlapped DEGs between db/db + ICS II versus db/db, and only 89 overlapped DEGs among the three groups. Moreover, hierarchical clustering analysis showed that the expression profiles of the DEGs in WT and db/db + ICS II groups were significantly different to that of the db/db group (Figure 3b). Furthermore, KEGG pathway analysis and enrichment of GO terms ascertained a considerable enrichment of genes involved in PPAR signaling pathway, insulin signaling pathway, and NF-κB signaling pathway (Figure 3c). The top 10 biological processes (BP), cellular components (CC), and molecular functions (MF) for up-regulated or down-regulated DEGs are listed in a bar chart (Figure 3d–f). 

### 3.4. ICS II Attenuated Oxidative Injury in db/db Mice

**Figure 4:**
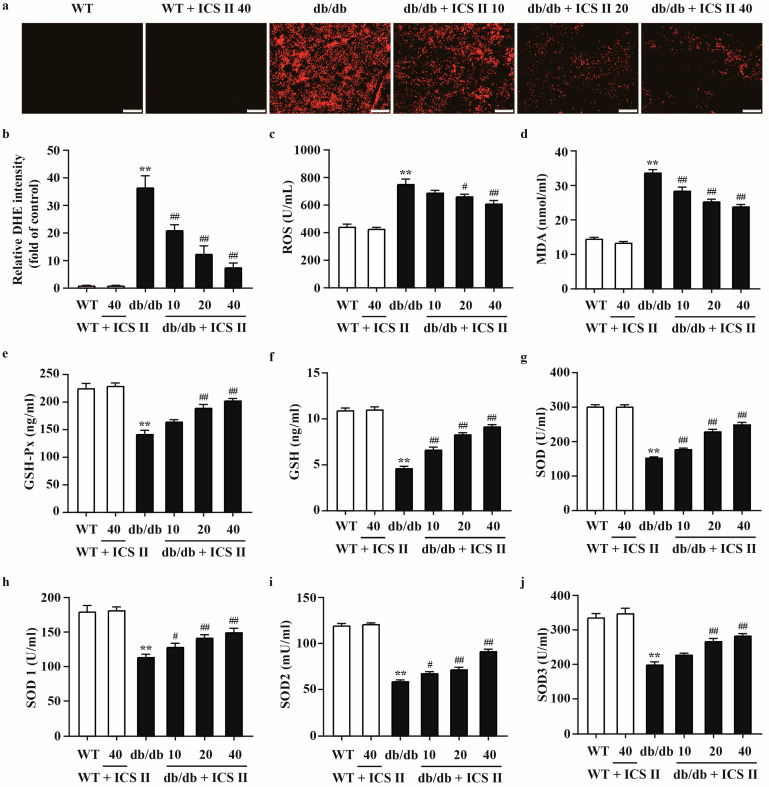
The effect of ICS II on oxidative injury in liver of db/db mice. (**a**) Representative DHE staining in the liver (×100, scale bar = 200 μm). (**b**) Quantitation of DHE staining. After the calculation of intensity per image, the signal intensity was divided by the captured area and expressed as folds of WT group (*n* = 5). (**c**) ROS level (*n* = 8). (**d**) MDA level (*n* = 8). (**e**) GSH-Px activity (*n* = 8). (**f**) GSH activity (*n* = 8). (**g**) SOD activity (*n* = 8). (**h**) SOD1 activity (*n* = 8). (**i**) SOD2 activity (*n* = 8). (**j**) SOD3 activity (*n* = 8). Data were expressed as mean ± SEM. ** *p* < 0.01 versus WT group; ^#^ *p* < 0.05 and ^##^ *p* < 0.01 versus db/db group.

To evaluate the effect of ICS II on oxidative stress in T2DM, the oxidative markers were determined using DHE staining and ELISA assay. The results showed that higher levels of DHE fluorescence intensity in livers and pancreas tissues of db/db mice compared with those of WT mice. ICS II evidently reduced DHE fluorescence intensity (Figure 4a,b and Supplementary Figure S2a,b). As shown in Figure 4c,d, ICS II also decreased the ROS and MDA levels in ICS II treated db/db mice than those of db/db mice. Moreover, ICS II significantly upregulated activities of GSH-Px (Figure 4e), GSH (Figure 4f), SOD (Figure 4g), SOD1 (Figure 4h), SOD2 (Figure 4i), and SOD3 (Figure 4j) in ICS II-treated db/db mice than those of db/db mice. These results indicate that ICS II attenuates oxidative injury in diabetic mice through inhibiting disequilibrium of redox status. 

### 3.5. ICS II Elevated the Protein Expressions of PPARα/γ and Inhibited NF-κB Signaling Pathway in db/db Mice

**Figure 5:**
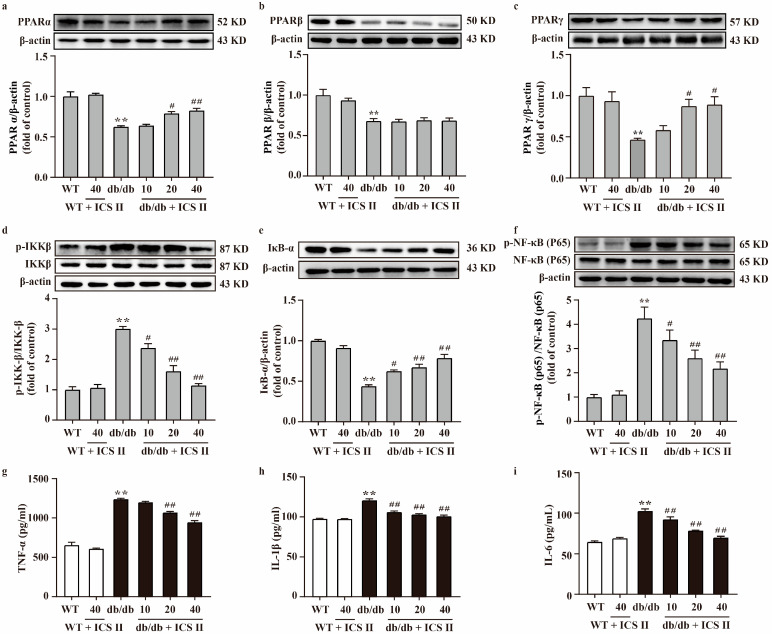
Effect of ICS II on the protein expressions of PPARα, PPARβ, PPARγ, and NF-κB signaling pathway in the liver of db/db mice. Representative western blot and quantitation of PPARα (**a**), PPARβ (**b**), and PPARγ (**c**) protein expressions in the liver (*n* = 5). (**d**) Representative western blot of phosphorylation of IKKβ protein level and quantitation of phosphorylation of IKKβ protein level in the liver. (**e**) Representative western blot of IκB-α protein expression and quantitation of IκB-α protein expression in the liver. (**f**) Representative western blot of phosphorylation of NF-κB (p65) protein level and quantitation of phosphorylation of NF-κB (p65) protein level in the liver (*n* = 5). Serum inflammatory factors were measured, including TNF-α (**g**), IL-1β (**h**), and IL-6 (**i**) (*n* = 8). Data were expressed as mean ± SEM. ** *p* < 0.01 versus WT group; ^#^ *p* < 0.05 and ^##^ *p* < 0.01 versus db/db group.

To further investigate the effect of ICS II on the PPAR and NF-κB signaling pathway, proteins extracted from the liver and pancreas were determined using western blot. The results showed that the protein expressions of PPARα, PPARβ, PPARγ, and IκB-α of liver and pancreas significantly decreased in db/db group compared with the WT group. Meanwhile, phosphorylation of NF-κB (p65) and IKKβ levels of liver and pancreas were markedly augmented in db/db group compared with the WT group. However, these changes were reversed by ICS II. Notably, ICS II did not affect the protein expression of PPARβ (Figure 5a–f and Supplementary Figure S3a–f). Additionally, the results indicated that TNF-α, IL-1β and IL-6 were significantly elevated in db/db group than those of WT group, whereas ICS II reduced these effects (Figure 5g–i). These findings support that the inhibitory effect of ICS II on inflammation of db/db mice, at least in part, through the activation of PPARα/γ.

### 3.6. ICS II Attenuated Insulin Resistance by Regulating the IRS1/Akt Signaling Transduction Pathway in db/db Mice

**Figure 6:**
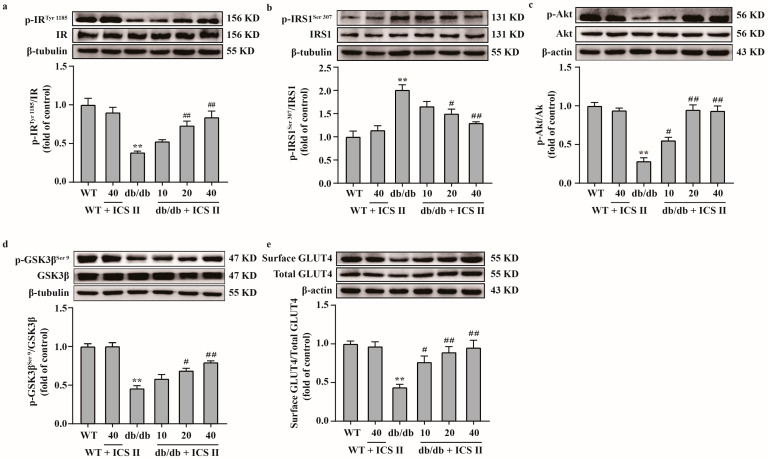
ICS II attenuated insulin resistance in db/db mice by regulating the IRS1/Akt signaling transduction pathway. (**a**) Representative western blot of phosphorylation level of IR^Tyr 1185^ and quantitation of phosphorylation level of IR^Tyr 1185^ in the liver (*n* = 5). (**b**) Representative western blot of phosphorylation level of IRS1^Ser 307^ and quantitation of phosphorylation level of IRS1^Ser 307^ in the liver (*n* = 5). (**c**) Representative western blot of phosphorylation level of Akt and quantitation of phosphorylation level of Akt in the liver (*n* = 5). (**d**) Representative western blot of phosphorylation level of GSK3β^Ser 9^ and quantitation of phosphorylation level of GSK3β^Ser 9^ in the liver (*n* = 5). (**e**) Representative western blot of cell surface and total GLUT4, and quantitation of cell surface GLUT4 content in the liver (*n* = 5). Data were expressed as mean ± SEM. ** *p* < 0.01 versus WT group; ^#^ *p* < 0.05 and ^##^ *p* < 0.01 versus db/db group.

Because the IRS1/Akt signaling transduction pathway plays a critical role in insulin resistance, we further elucidated the effect of ICS II on the IRS1/Akt signaling transduction pathway. The results showed that the phosphorylation levels of IR^Tyr 1185^, Akt and GSK3β^Ser 9^ were significantly decreased and the phosphorylation level of IRS1^Ser 307^ was increased in db/db group compared with the WT group. However, ICS II significantly reversed these changes (Figure 6a–d). Moreover, the results showed that the expression of surface GLUT4 protein in liver tissue was increased in ICS II treated db/db mice than those of db/db mice, which indicated that ICS II promoted GLUT4 translocation in liver (Figure 6e). These findings suggest that ICS II attenuated insulin resistance by regulating IRS1/Akt signaling transduction pathway.

### 3.7. ICS II Protected against PA-Induced Injury in HepG2 Cells and MIN6 Cells via Upregulating the Protein Expressions of PPARα/γ, and Inhibited NF-κB Signaling Pathway

**Figure 7:**
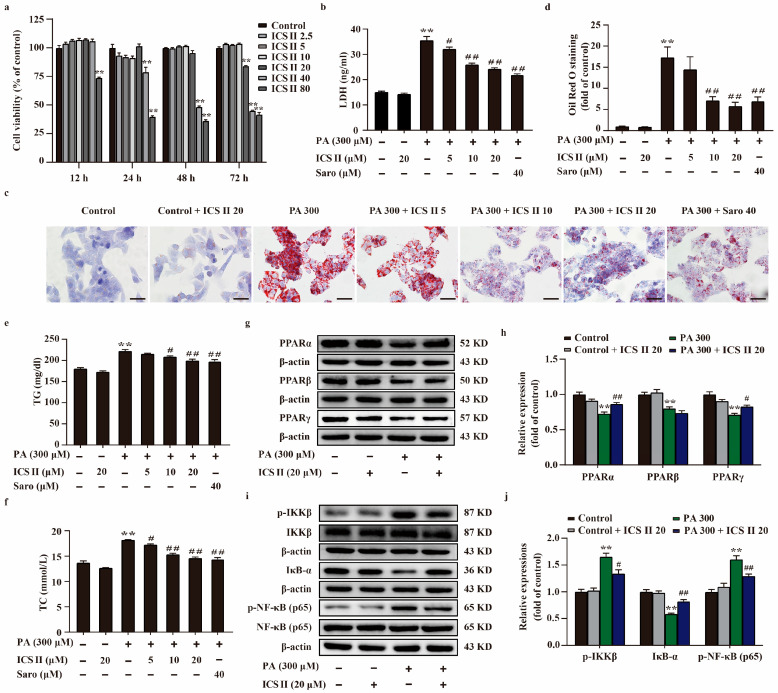
ICS II protected against PA-induced lipid accumulation and cytotoxicity in HepG2 cells via upregulating PPARα/γ protein expressions and inhibiting NF-κB signaling pathway. (**a**) The safe concentration range of ICS II within 12 h, 24 h, 48 h, and 72 h (*n* = 6). (**b**) Cytotoxicity was determined by the LDH release assay (*n* = 6). (**c**) Oil red O staining of HepG2 cells (×400, scale bar = 50 μm). (**d**) Quantitative analysis of Oil red O staining in HepG2 cells (*n* = 5). TG (**e**) and TC (**f**) levels in HepG2 cells (*n* = 6). (**g**) Representative western blot of PPARα, PPARβ, and PPARγ protein expressions. (**h**) Quantitation of PPARα, PPARβ, and PPARγ protein expressions (*n* = 5). (**i**) Representative western blot of IκB-α protein expression, phosphorylation of level IKKβ and NF-κB (p65). (**j**) Quantitation of IκB protein expression, phosphorylation level of IKKβ and NF-κB (p65) (*n* = 5). Data were expressed as mean ± SEM. ** *p* < 0.01 versus control group; ^#^ *p* < 0.05 and ^##^ *p* < 0.01 versus PA 300 group.

To further explore the role of ICS II during T2DM, HepG2 cells and MIN6 cells were applied to mimic T2DM by stimulation of the cells with PA. The results showed that ICS II exerted no effect on the cell viability HepG2 cells and MIN6 cells below 20 μM within 24 h (Figure 7a and Supplementary Figure S4a), as evidenced by MTT assay. In parallel, ICS II reduced the amount of LDH release following PA insult HepG2 cells (Figure 7b). As shown in Figure 7c–f, more lipid droplets were observed in PA-induced HepG2 cells than those of the control group, and the intracellular TC and TG levels were significantly increased in PA-induced HepG2 cells in comparison with the control group. However, ICS II reversed this change. The results also showed that ICS II protected against PA-induced MIN6 cells injury in a concentration-dependent manner (Supplementary Figure S4b). Additionally, PA resulted in MIN6 cells shrink, depletion in cell numbers, even death. ICS II reversed these changes after PA treatment, as evidenced by observation of light converted microscopy (Supplementary Figure S4c). To further verify our results, we sequentially applied western blot to examine the protein expression levels of PPARα, PPARβ, PPARγ, and NF-κB signaling in HepG2 cells and MIN6 cells. The results showed that the expressions of PPARα, PPARβ, and PPARγ were decreased following PA, however, ICS II reversed these effects of PPARα and PPARγ, but had no effect on PPARβ (Figure 7g,h, and Supplementary Figure S4d,e). Meanwhile, phosphorylation levels of NF-κB (p65) and IKKβ were markedly augmented following PA than those of control group, whereas ICS II reduced these effects. In contrast, ICS II significantly increased IκB-α protein expression than that of PA-treated group (Figure 7i,j and Supplementary Figure S4f,g). These data show that ICS II protects against PA-induced injury in HepG2 cells and MIN6 cells via mediating the protein expressions of PPARα/γ/NF-κB signaling pathway.

### 3.8. ICS II Mitigated Oxidative Stress in PA-Induced HepG2 Cells

**Figure 8:**
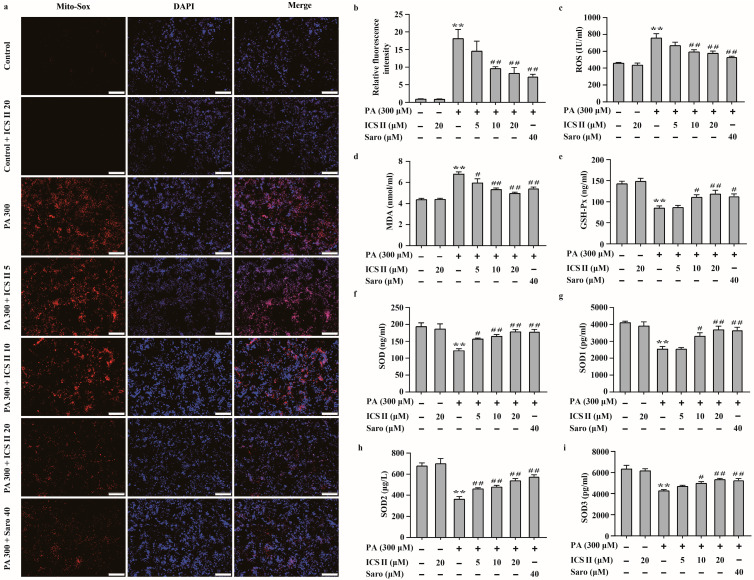
ICS II mitigated oxidative stress in PA-induced HepG2 cells. (**a**) Mito-SOX staining of HepG2 cells (×100, scale bar = 200 μm). (**b**) Relative fluorescence intensity (*n* = 5). (**c**) ROS level (*n* = 6). (**d**) MDA level (*n* = 6). (**e**) GSH-Px activity (*n* = 6). (**f**) SOD activity (*n* = 6). (**g**) SOD1 activity (*n* = 6). (**h**) SOD2 activity (*n* = 6). (**i**) SOD3 activity (*n* = 6). Data were expressed as mean ± SEM. ** *p* < 0.01 versus control group; ^#^ *p* < 0.05 and ^##^ *p* < 0.01 versus PA 300 group.

The effect of ICS II on PA-induced oxidative stress in HepG2 cells and MIN6 cells were also investigated. We observed that the intracellular O^2^^⋅−^ was augmented in PA treated group compared to that of control group, as evidenced by Mito-SOX staining. However, ICS II reduced these effects (Figure 8a,b). The results also showed that the levels of oxidative stress markers, including ROS and MDA, were significantly higher in PA-treated group than those of control group, while ICS II prominently reversed these changes (Figure 8c,d). Moreover, activities of the antioxidant markers including GSH-Px, SOD, and its subtypes SOD1, SOD2, and SOD3 were lower in PA-treated group than those of control group, while ICS II apparently reversed these changes (Figure 8e–i). These findings suggest that ICS II mitigates PA-induced oxidative stress in HepG2 cells.

### 3.9. ICS II Attenuated Insulin Resistance in PA-Induced HepG2 Cells by IRS1/Akt Signaling Transduction Pathway

**Figure 9:**
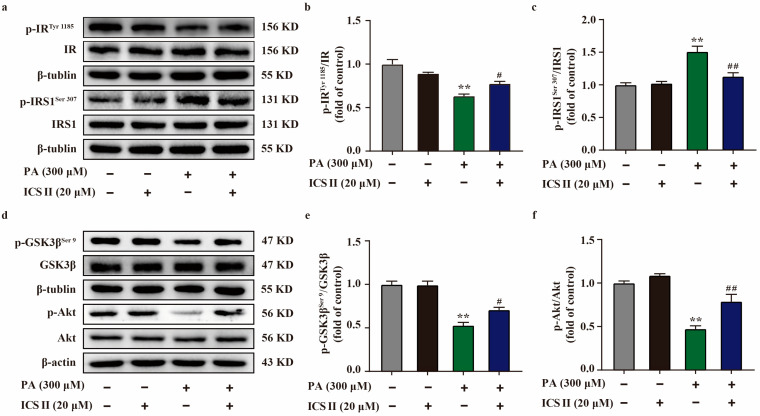
ICS II attenuated insulin resistance in PA-induced HepG2 cells by regulating the IRS1/Akt signaling transduction pathway. (**a**) Representative western blot of phosphorylation level of IR^Tyr 1185^ and IRS1^Ser 307^. (**b**) Quantitation of phosphorylation level of IR^Tyr 1185^ (*n* = 5). (**c**) Quantitation of phosphorylation level of IRS1^Ser 307^ (*n* = 5). (**d**) Representative western blot of phosphorylation level of GSK3β^Ser 9^ and Akt. (**e**) Quantitation of phosphorylation level of GSK3β^Ser 9^. (**f**) Quantitation of phosphorylation level of Akt (*n* = 5). Data were expressed as mean ± SEM. ** *p* < 0.01 versus control group; ^#^ *p* < 0.05 and ^##^ *p* < 0.01 versus PA 300 group.

To further verify our results in vivo, we sequentially applied western blot to examine the protein expression levels related to IRS1/Akt signaling transduction pathway in PA-induced HepG2 cells. As shown in Figure 9a–f, the phosphorylation levels of IR^Tyr 1185^, Akt, and GSK3β^Ser 9^ were significantly decreased in PA-induced HepG2 cells compared with the control group. However, ICS II markedly reversed these changes. Moreover, ICS II also attenuated the increase in phosphorylation level of IRS1^Ser 307^ after PA insult, which is consistent with the results in vivo. These findings confirm that ICS II attenuated insulin resistance, at least partly, through IRS1/Akt signaling transduction pathway.

### 3.10. Effect of ICS II on PA-Induced Injury in PPARα/γ-KO HepG2 Cells and MIN6 Cells

**Figure 10:**
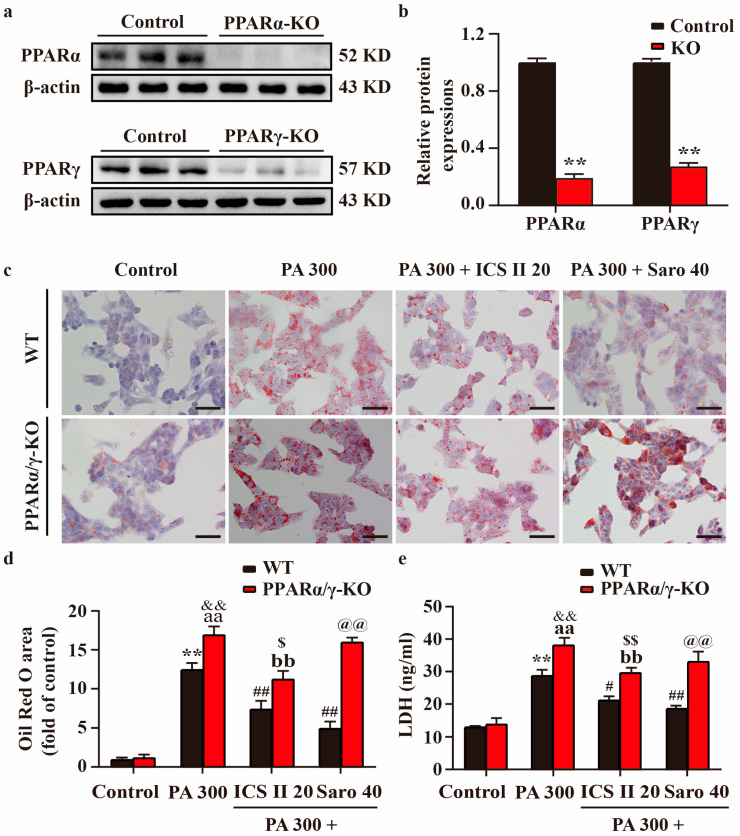
Effect of ICS II on PA-induced injury in PPARα/γ-KO HepG2 cells. (**a**) Representative western blot of PPARα and PPARγ protein expressions after knockout of PPARα/γ. (**b**) Quantitation of PPARα/γ protein expressions (*n* = 6). (**c**) Oil red O staining of HepG2 cells (×400, scale bar = 50 μm). Treatment with or without ICS II (20 μM) in PPARα/γ-KO HepG2 cells and WT HepG2 cells. (**d**) Quantitation of Oil red O staining (*n* = 5). (**e**) LDH level. Treatment with or without ICS II (20 μM) in PPARα/γ-KO HepG2 cells and WT HepG2 cells. (*n* = 6). Data were expressed as mean ± SEM. ** *p* < 0.01 versus control group (WT); ^#^ *p* < 0.05 and ^##^ *p* < 0.01 versus PA 300 group (WT); ^aa^ *p* < 0.01 versus control group (PPARα/γ-KO); ^bb^ *p* < 0.01 versus PA 300 group (PPARα/γ-KO); ^&&^ *p* < 0.01 versus PA 300 group (WT); ^$^ *p* < 0.05 and ^$$^ *p* < 0.01 versus PA 300 + ICS II 20 group (WT); ^@@^ *p* < 0.01 versus PA 300 + Saro 40 group (WT).

PPARα/γ-KO HepG2 cells and MIN6 cells were generated using the CRISPR/Cas9 system, which resulted in > 70% loss in PPARα/γ protein levels relative to control cells (Figure 10a,b, and Supplementary Figure S5a,b). The results showed that PPARα/γ-KO HepG2 cells and MIN6 cells showed more severe injury in PA-treated group than that of control group. However, the protective effects of ICS II or Saro on PA-induced injury were partially abolished in HepG2 cells and MIN6 cells (Figure 10c–e and Supplementary Figure S5c,d). These findings highlight that ICS II-mediated protection on PA-induced injury is, at least partly, dependent on the presence of PPARα/γ.

## 4. Discussion

**Figure 11:**
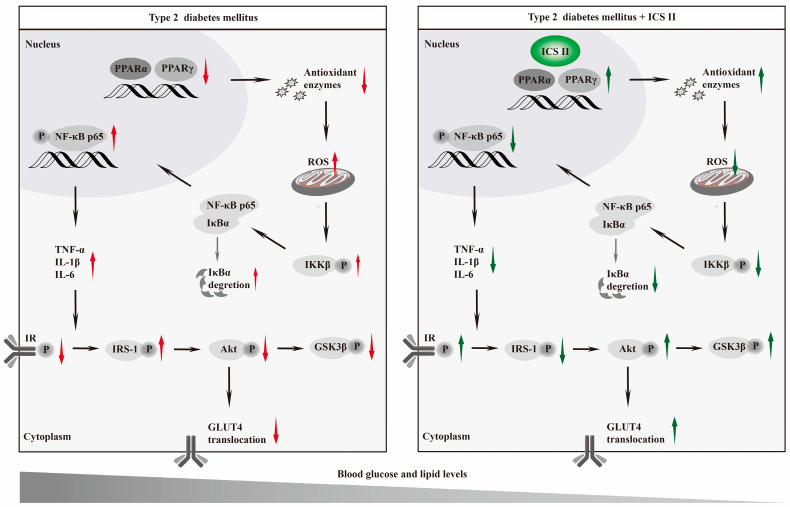
Graphic abstract Schematic depicting the role of ICS II on T2DM. ICS II attenuated hyperglycemia and hyperlipidemia by targeting PPARα/γ: involvement of ROS/NF-κB/IRS1 signaling pathway, leading to reduced levels of inflammatory factors and oxidative stress, as well as alleviation of insulin resistance.

The present study, for the first time, discovered that: (1) ICS II, a naturally occurring flavonoids compound, effectively overcomes T2DM; (2) ICS II protected against T2DM due to attenuate oxidative stress and inflammation; (3) The exciting therapeutic effect of ICS II on T2DM might be mediated by targeting PPARα/γ (Figure 11). 

In this study, we firstly explored the therapeutic potential of ICS II using db/db mice. Interestingly, the present study indicated that ICS II effectively combats T2DM, as evidenced by reducing hyperglycemia, concurrently enhanced glucose tolerance, and insulin sensitivity in db/db mice. Apart from the imbalance in glucose metabolism, dyslipidemia usually co-exist during T2DM [29,30]. Thus, the effect of ICS II on lipid metabolism were explored, and we found that ICS II efficiently regulated lipid metabolism and reversed the excessive accumulation of fat in liver of db/db mice. In addition, glucose and lipid abnormalities are important factors, resulting in the progression of β cells dysfunction or pancreatic islets destruction [31,32,33]. Our findings indicated that ICS II significantly improved structure of pancreas and restored the numbers of β cells in db/db mice, which further confirmed that ICS II was potently against T2DM, and its possible underlying mechanism is worth investigating in depth. Interestingly, insulin signaling pathway, PPARs signaling pathways, and NF-κB signaling pathway were discovered in the therapeutic effect of ICS II on T2DM, as evidenced by transcriptome analysis. 

Insulin resistance is mediated by impaired insulin signaling, particularly disruption of the insulin receptor substrate1 (IRS1)/Akt pathway, which leads to insulin insensitivity and glucose intolerance [34,35,36]. In a normal condition, insulin binds to the insulin receptor to activate its intrinsic receptor tyrosine kinase, resulting in tyrosine residues phosphorylation of IRS1, which in turn initiate Akt, one of the major indicators in the insulin signaling pathway [37]. Once activated, Akt phosphorylates its downstream glycogen synthase kinase 3β (GSK-3β) to increase glycogen synthesis and maintains lipid homeostasis [38]. Moreover, the insulin-stimulated phosphorylation of Akt is crucial to insulin-responsive GLUT, which is from cytosol to the cell membrane to facilitate glucose transport into cells [39]. Among the GLUT subtypes, GLUT4 is mainly enriched in the liver, pancreas, and skeletal muscle [40]. During the development of T2DM, disruption of insulin signaling results in decrease of hepatic GSK-3β, along with impairment of GLUT4 translocation [41,42]. Our findings revealed that ICS II not only effectively promoted phosphorylation of GSK-3β, but also facilitated GLUT4 translocated onto the cell membrane and increase glucose uptake. These findings suggest that the IRS1/Akt/GSK-3β/GLUT4 signaling pathway, at least partly, contributes the beneficial effect of ICS II on hepatic glycogenesis and insulin resistance in T2DM.

Accumulating evidence suggests that oxidative stress and inflammation play major roles in the development of T2DM [43,44]. Under normal physiological conditions, the production of ROS and elimination of ROS by antioxidant enzymes (such as catalase, SOD, and GSH-Px) are balanced to maintain the redox status. Under T2DM stimuli, the redox equilibrium is broken and excessive ROS impairs lipid and glucose metabolism, leading to hepatic steatosis, insulin resistance, and hyperglycemia [45]. Additionally, excessive ROS also provoke the activation of NF-κB signaling and inflammatory cascade to orchestrate the occurrence of T2DM. Under resting state, NF-κB (p50/p65) heterodimers are bound to IκB-α in the cytoplasm. Upon excessive ROS stimuli, IKK was activated and subsequently phosphorylates and degraded the IκB-α, which facilitates NF-κB translocates into the nucleus and initiates the release of proinflammatory cytokines [46]. Our findings demonstrated that ICS II significantly restored the redox balance and inhibited inflammatory response through mediation of NF-κB signaling pathway. 

However, the detail mechanism of ICS II on T2DM is still unclear. Based on these findings mentioned above, we assumed that PPARα/γ might be the potential targets of ICS II to overcome T2DM. To test our hypothesis, we further observed the effect of ICS II on PPARα/γ in T2DM both in vivo and in vitro. The findings in the present study clearly demonstrated that ICS II markedly up-regulated the protein expressions of PPARα/γ both in the liver and pancreas tissues of db/db mice, as well as in PA-induced injury of HepG2 cells and MIN6 cells. Intriguingly, we further found that PPARα/γ deficiency, to some extent, abolished the protective effect of ICS II on PA-induced injury in HepG2 cells and MIN6 cells, as evidenced by CRISPR-Cas9 system. These findings confirm that PPARα/γ might be the potential targets of ICS II to treat T2DM. 

In spite of the encouraging experimental evidence, there are still limitations in the current study. Firstly, whether ICS II can develop a novel naturally occurring dual PPARα/γ agonist still requires further exploration. Secondly, the precise mechanism of ICS II against T2DM and preventive therapeutic efficacy of ICS II on T2DM is worth exploring in-depth. Thirdly, female and male cells respond differently to chemical and microbial stressors, and efficacy is also affected in animals of different sexes [47]. These factors will be systematically considered in our subsequent studies. Finally, previous studies have reported the effectiveness of ICS II against diabetic complications. Whether ICS II can conquer T2DM and treat diabetic complications is a mystery. 

## 5. Conclusions

To summarize, ICS II exerts potent anti-T2DM effect with anti-oxidative and anti-inflammatory properties, at least partly by targeting PPARα/γ-mediated ROS/NF-κB/IRS1 signaling pathway.

## Figures and Tables

**Table 1:** The PPARα/γ gRNA sequences.

Gene	gRNA Sequences
PPARα	CACCGCATCTGTCCTCTCTCCCCAC AAAC GTGGGGAGAGAGGACAGATG
PPARγ	CACCGCAACTTCGGAATCAGCTCTG AAACCAGAGCTGATTCCGAAGTTG

## Data Availability

All data related to this research are presented in the manuscript.

## Supplementary Materials

The following supporting information can be downloaded at: https://www.mdpi.com/article/10.3390/antiox11091705/s1, Figure S1: Effect of ICS II on body weight and liver weight in db/db mice; Figure S2: Effect of ICS II on oxidative injury in pancreas of db/db mice; Figure S3: Effect of ICS II on the protein expressions of PPARα, PPARβ, PPARγ and NF-κB signaling pathway in pancreas of db/db mice; Figure S4: ICS II protected against PA-induced injury in MIN6 cells via upregulating PPARα/γ protein expressions and inhibiting NF-κB signaling pathway; Figure S5: Effect of ICS II on PA-induced injury in PPARα/γ-KO MIN6 cells.

## References

[1] Chatterjee S., Davies M.J., Heller S., Speight J., Snoek F.J., Khunti K. (2018). Diabetes structured self-management education programmes: A narrative review and current innovations. Lancet Diabetes Endocrinol..

[2] Ali M.K., Pearson-Stuttard J., Selvin E., Gregg E.W. (2022). Interpreting global trends in type 2 diabetes complications and mortality. Diabetologia.

[3] Quattrocchi E., Goldberg T., Marzella N. (2020). Management of type 2 diabetes: Consensus of diabetes organizations. Drugs Context.

[4] Stein S.A., Lamos E.M., Davis S.N. (2013). A review of the efficacy and safety of oral antidiabetic drugs. Expert Opin. Drug Saf..

[5] Yang Q., Vijayakumar A., Kahn B.B. (2018). Metabolites as regulators of insulin sensitivity and metabolism. Nat. Rev. Mol. Cell Biol..

[6] Burgos-Moron E., Abad-Jimenez Z., Maranon A.M., Iannantuoni F., Escribano-Lopez I., Lopez-Domenech S., Salom C., Jover A., Mora V., Roldan I. (2019). Relationship Between Oxidative Stress, ER Stress, and Inflammation in Type 2 Diabetes: The Battle Continues. J. Clin. Med..

[7] Alu S.N., Los E.A., Ford G.A., Stone W.L. (2022). Oxidative Stress in Type 2 Diabetes: The Case for Future Pediatric Redoxomics Studies. Antioxidants.

[8] Houstis N., Rosen E.D., Lander E.S. (2006). Reactive oxygen species have a causal role in multiple forms of insulin resistance. Nature.

[9] Tian Y., Li Y., Liu J., Lin Y., Jiao J., Chen B., Wang W., Wu S., Li C. (2022). Photothermal therapy with regulated Nrf2/NF-kappaB signaling pathway for treating bacteria-induced periodontitis. Bioact. Mater..

[10] Yuan Y.L., Lin B.Q., Zhang C.F., Cui L.L., Ruan S.X., Yang Z.L., Li F., Ji D. (2016). Timosaponin B-II Ameliorates Palmitate-Induced Insulin Resistance and Inflammation via IRS-1/PI3K/Akt and IKK/NF-κB Pathways. Am. J. Chin. Med..

[11] Jha J.C., Ho F., Dan C., Jandeleit-Dahm K. (2018). A causal link between oxidative stress and inflammation in cardiovascular and renal complications of diabetes. Clin. Sci..

[12] Venteclef N., Jakobsson T., Steffensen K.R., Treuter E. (2011). Metabolic nuclear receptor signaling and the inflammatory acute phase response. Trends Endocrinol. Metab..

[13] Gross B., Pawlak M., Lefebvre P., Staels B. (2017). PPARs in obesity-induced T2DM, dyslipidaemia and NAFLD. Nat. Rev. Endocrinol..

[14] Zhang C., Deng J., Liu D., Tuo X., Xiao L., Lai B., Yao Q., Liu J., Yang H., Wang N. (2018). Nuciferine ameliorates hepatic steatosis in high-fat diet/streptozocin-induced diabetic mice through a PPARalpha/PPARgamma coactivator-1alpha pathway. Br. J. Pharmacol..

[15] Dubois V., Eeckhoute J., Lefebvre P., Staels B. (2017). Distinct but complementary contributions of PPAR isotypes to energy homeostasis. J. Clin. Investig..

[16] Rigano D., Sirignano C., Taglialatela-Scafati O. (2017). The potential of natural products for targeting PPARalpha. Acta Pharm. Sin. B.

[17] Pawlak M., Lefebvre P., Staels B. (2015). Molecular mechanism of PPARalpha action and its impact on lipid metabolism, inflammation and fibrosis in non-alcoholic fatty liver disease. J. Hepatol..

[18] Leonardini A., Laviola L., Perrini S., Natalicchio A., Giorgino F. (2009). Cross-Talk between PPARgamma and Insulin Signaling and Modulation of Insulin Sensitivity. PPAR Res..

[19] Bortolini M., Wright M.B., Bopst M., Balas B. (2013). Examining the safety of PPAR agonists-current trends and future prospects. Expert Opin. Drug Saf..

[20] Feng L., Luo H., Xu Z., Yang Z., Du G., Zhang Y., Yu L., Hu K., Zhu W., Tong Q. (2016). Bavachinin, as a novel natural pan-PPAR agonist, exhibits unique synergistic effects with synthetic PPAR-gamma and PPAR-alpha agonists on carbohydrate and lipid metabolism in db/db and diet-induced obese mice. Diabetologia.

[21] Sun Y.S., Thakur K., Hu F., Zhang J.G., Wei Z.J. (2020). Icariside II inhibits tumorigenesis via inhibiting AKT/Cyclin E/CDK2 pathway and activating mitochondria-dependent pathway. Pharmacol. Res..

[22] Deng Y., Long L., Wang K., Zhou J., Zeng L., He L., Gong Q. (2017). Icariside II, a Broad-Spectrum Anti-cancer Agent, Reverses Beta-Amyloid-Induced Cognitive Impairment through Reducing Inflammation and Apoptosis in Rats. Front. Pharmacol..

[23] Zheng Y., Deng Y., Gao J.M., Lv C., Lang L.H., Shi J.S., Yu C.Y., Gong Q.H. (2020). Icariside II inhibits lipopolysaccharide-induced inflammation and amyloid production in rat astrocytes by regulating IKK/IkappaB/NF-kappaB/BACE1 signaling pathway. Acta Pharmacol. Sin..

[24] Gao J., Deng Y., Yin C., Liu Y., Zhang W., Shi J., Gong Q. (2017). Icariside II, a novel phosphodiesterase 5 inhibitor, protects against H2O2-induced PC12 cells death by inhibiting mitochondria-mediated autophagy. J. Cell. Mol. Med..

[25] Feng L., Gao J., Liu Y., Shi J., Gong Q. (2018). Icariside II alleviates oxygen-glucose deprivation and reoxygenation-induced PC12 cell oxidative injury by activating Nrf2/SIRT3 signaling pathway. Biomed. Pharmacother..

[26] Yin C., Deng Y., Liu Y., Gao J., Yan L., Gong Q. (2018). Icariside II Ameliorates Cognitive Impairments Induced by Chronic Cerebral Hypoperfusion by Inhibiting the Amyloidogenic Pathway: Involvement of BDNF/TrkB/CREB Signaling and Up-Regulation of PPARalpha and PPARgamma in Rats. Front. Pharmacol..

[27] Deng Y., Xiong D., Yin C., Liu B., Shi J., Gong Q. (2016). Icariside II protects against cerebral ischemia-reperfusion injury in rats via nuclear factor-kappaB inhibition and peroxisome proliferator-activated receptor up-regulation. Neurochem. Int..

[28] Shi Y.L., Zhang Y.P., Luo H., Xu F., Gao J.M., Shi J.S., Gong Q.H. (2022). Trilobatin, a Natural Food Additive, Exerts Anti-Type 2 Diabetes Effect Mediated by Nrf2/ARE and IRS-1/GLUT2 Signaling Pathways. Front. Pharmacol..

[29] Bril F., Cusi K. (2017). Management of Nonalcoholic Fatty Liver Disease in Patients with Type 2 Diabetes: A Call to Action. Diabetes Care.

[30] Johnson J.D. (2021). On the causal relationships between hyperinsulinaemia, insulin resistance, obesity and dysglycaemia in type 2 diabetes. Diabetologia.

[31] Hu L., Zhou Z., Deng L., Ren Q., Cai Z., Wang B., Li Z., Wang G. (2020). HWL-088, a new and highly effective FFA1/PPARdelta dual agonist, attenuates nonalcoholic steatohepatitis by regulating lipid metabolism, inflammation and fibrosis. J. Pharm. Pharmacol..

[32] Halban P.A., Polonsky K.S., Bowden D.W., Hawkins M.A., Ling C., Mather K.J., Powers A.C., Rhodes C.J., Sussel L., Weir G.C. (2014). beta-cell failure in type 2 diabetes: Postulated mechanisms and prospects for prevention and treatment. Diabetes Care.

[33] Wang P., Fiaschi-Taesch N.M., Vasavada R.C., Scott D.K., Garcia-Ocana A., Stewart A.F. (2015). Diabetes mellitus--advances and challenges in human beta-cell proliferation. Nat. Rev. Endocrinol..

[34] Zheng T., Yang X., Wu D., Xing S., Bian F., Li W., Chi J., Bai X., Wu G., Chen X. (2015). Salidroside ameliorates insulin resistance through activation of a mitochondria-associated AMPK/PI3K/Akt/GSK3beta pathway. Br. J. Pharmacol..

[35] Zhang L., Li X., Zhang N., Yang X., Hou T., Fu W., Yuan F., Wang L., Wen H., Tian Y. (2020). WDFY2 Potentiates Hepatic Insulin Sensitivity and Controls Endosomal Localization of the Insulin Receptor and IRS1/2. Diabetes.

[36] Song B.-R., Alam M.B., Lee S.-H. (2022). Terpenoid-Rich Extract of Dillenia indica L. Bark Displays Antidiabetic Action in Insulin-Resistant C2C12 Cells and STZ-Induced Diabetic Mice by Attenuation of Oxidative Stress. Antioxidants.

[37] Hancock M.L., Meyer R.C., Mistry M., Khetani R.S., Wagschal A., Shin T., Ho Sui S.J., Naar A.M., Flanagan J.G. (2019). Insulin Receptor Associates with Promoters Genome-wide and Regulates Gene Expression. Cell.

[38] Chakraborty A., Koldobskiy M.A., Bello N.T., Maxwell M., Potter J.J., Juluri K.R., Maag D., Kim S., Huang A.S., Dailey M.J. (2010). Inositol pyrophosphates inhibit Akt signaling, thereby regulating insulin sensitivity and weight gain. Cell.

[39] Zhu Y., Jing L., Li X., Zheng D., Zhou G., Zhang Y., Sang Y., Shi Z., Sun Z., Zhou X. (2021). Decabromodiphenyl ether disturbs hepatic glycolipid metabolism by regulating the PI3K/AKT/GLUT4 and mTOR/PPARgamma/RXRalpha pathway in mice and L02 cells. Sci. Total Environ..

[40] Sunil C., Irudayaraj S.S., Duraipandiyan V., Alrashood S.T., Alharbi S.A., Ignacimuthu S. (2021). Friedelin exhibits antidiabetic effect in diabetic rats via modulation of glucose metabolism in liver and muscle. J. Ethnopharmacol..

[41] Gandhi G.R., Stalin A., Balakrishna K., Ignacimuthu S., Paulraj M.G., Vishal R. (2013). Insulin sensitization via partial agonism of PPARgamma and glucose uptake through translocation and activation of GLUT4 in PI3K/p-Akt signaling pathway by embelin in type 2 diabetic rats. Biochim. Biophys. Acta.

[42] Luo P., Zheng M., Zhang R., Zhang H., Liu Y., Li W., Sun X., Yu Q., Tipoe G.L., Xiao J. (2021). S-Allylmercaptocysteine improves alcoholic liver disease partly through a direct modulation of insulin receptor signaling. Acta Pharm. Sin. B.

[43] Yu Y.Y., Cui S.C., Zheng T.N., Ma H.J., Xie Z.F., Jiang H.W., Li Y.F., Zhu K.X., Huang C.G., Li J. (2021). Sarsasapogenin improves adipose tissue inflammation and ameliorates insulin resistance in high-fat diet-fed C57BL/6J mice. Acta Pharmacol. Sin..

[44] Carvalho C., Cardoso S. (2021). Diabetes-Alzheimer’s Disease Link: Targeting Mitochondrial Dysfunction and Redox Imbalance. Antioxid. Redox Signal..

[45] Lennicke C., Cocheme H.M. (2021). Redox regulation of the insulin signalling pathway. Redox Biol..

[46] Arkan M.C., Hevener A.L., Greten F.R., Maeda S., Li Z.W., Long J.M., Wynshaw-Boris A., Poli G., Olefsky J., Karin M. (2005). IKK-beta links inflammation to obesity-induced insulin resistance. Nat. Med..

[47] Collins F.S., Clayton J.A. (2014). NIH to balance sex in cell and animal studies. Nature.

