# Supplementary figures and images for: Icariside II Exerts Anti-Type 2 Diabetic Effect by Targeting PPARα/γ: Involvement of ROS/NF-κB/IRS1 Signaling Pathway
Source: Antioxidants (Basel). 2022 Aug 30;11(9):1705. doi: 10.3390/antiox11091705 (PMC9495514; doi:10.3390/antiox11091705)

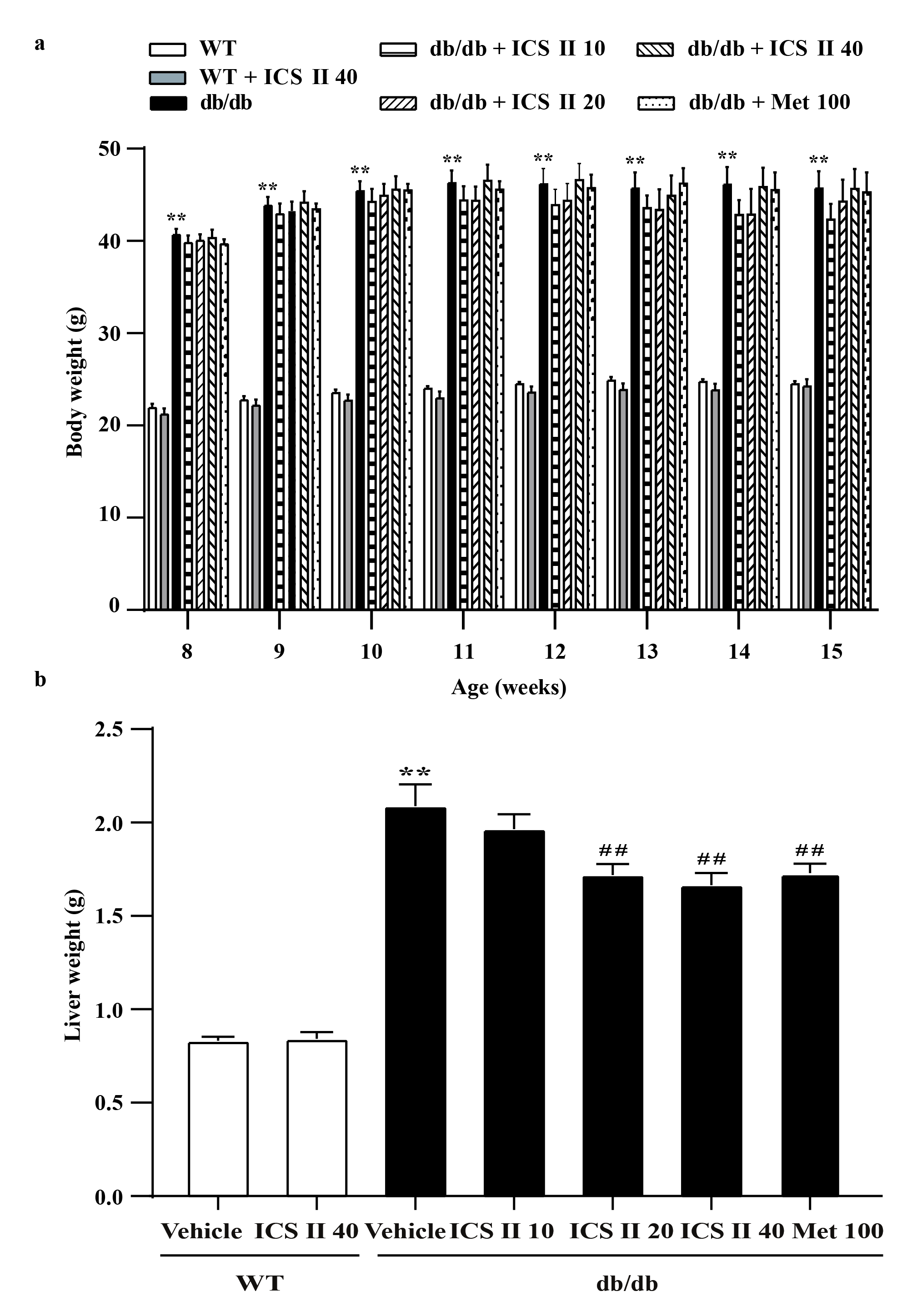

Supplement: Supplementary file 1 [file antioxidants-11-01705-s001.zip › supplementary Figures/Supplementary Fig. 1.tif]

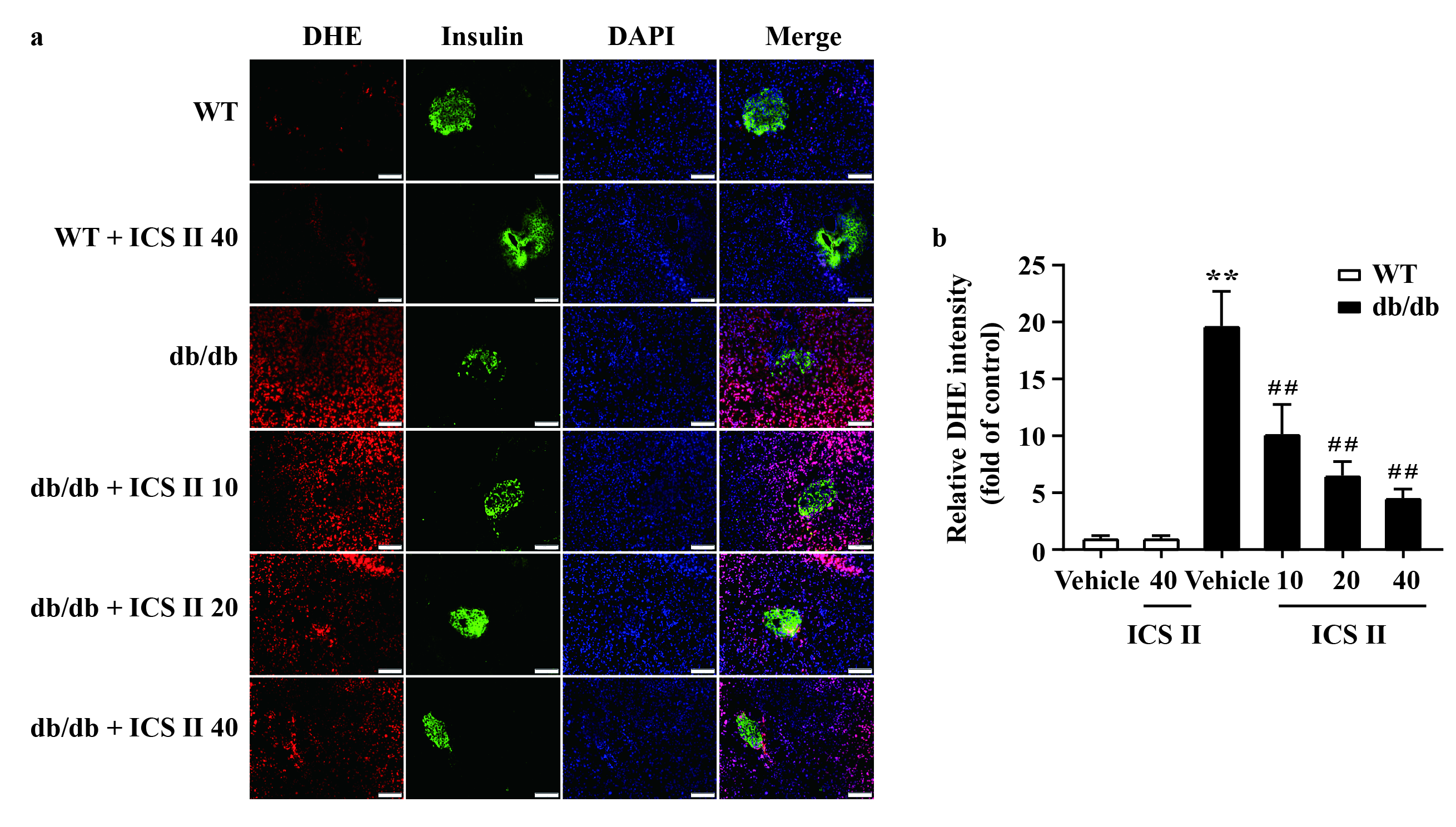

Supplement: Supplementary file 1 [file antioxidants-11-01705-s001.zip › supplementary Figures/Supplementary Fig. 2.tif]

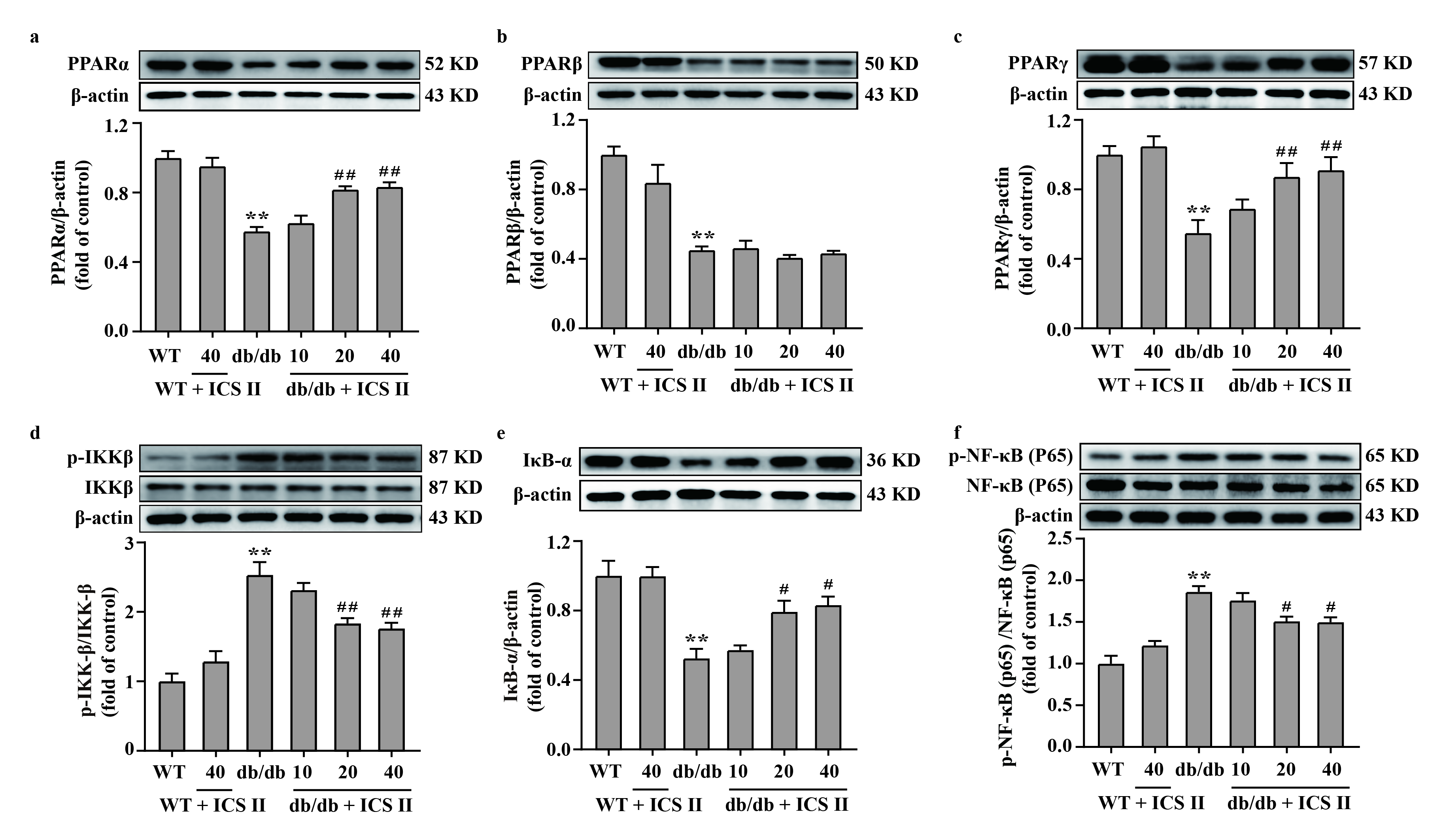

Supplement: Supplementary file 1 [file antioxidants-11-01705-s001.zip › supplementary Figures/Supplementary Fig. 3.tif]

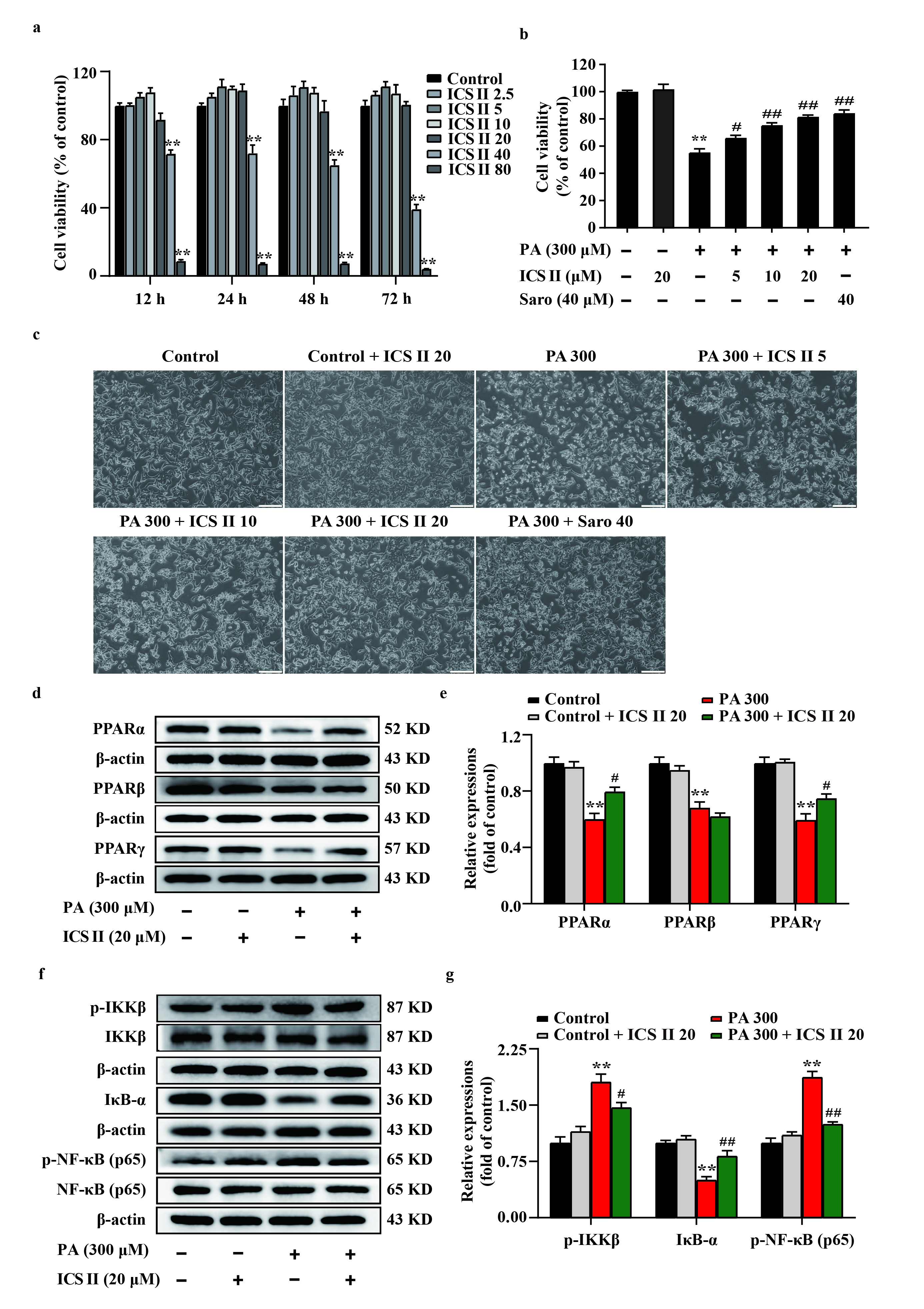

Supplement: Supplementary file 1 [file antioxidants-11-01705-s001.zip › supplementary Figures/Supplementary Fig. 4.tif]

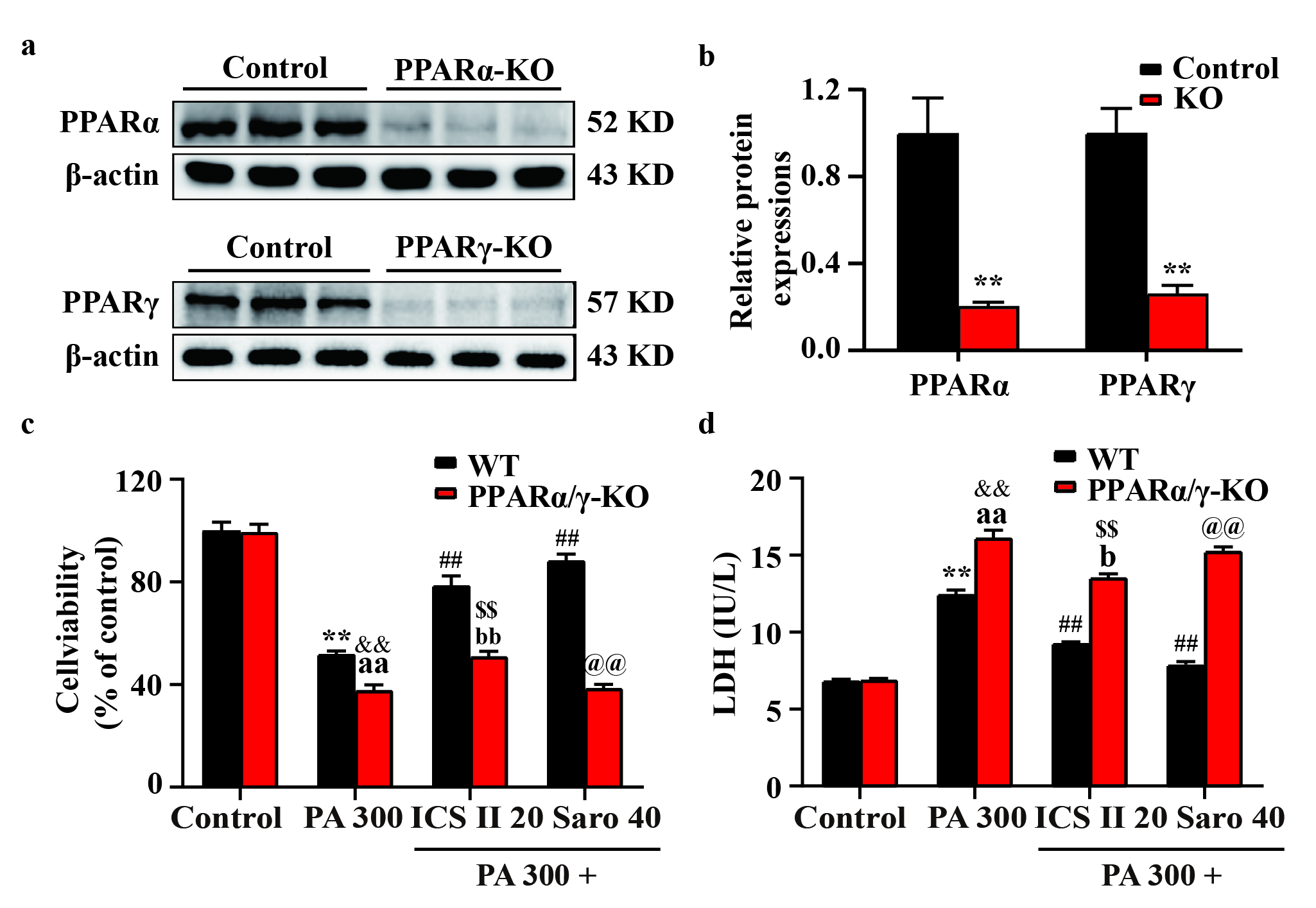

Supplement: Supplementary file 1 [file antioxidants-11-01705-s001.zip › supplementary Figures/Supplementary Fig. 5.tif]
